# Clinical Evaluation of Doppler Blood Pressure Measurement in Continuous-Flow LVAD Patients: Implications for Postoperative Management

**DOI:** 10.3390/jcdd13060276

**Published:** 2026-06-18

**Authors:** Umit Kahraman, Emrah Oguz, Vusali Kasumovi, Aysen Yaprak Kapkin, Ahmet Daylan, Serkan Ertugay, Sanem Nalbantgil, Cagatay Engin, Mustafa Ozbaran, Tahir Yagdi

**Affiliations:** 1 Department of Cardiovascular Surgery, Faculty of Medicine, Ege University, 35100 Izmir, Turkey; umit.kahraman@ege.edu.tr (U.K.); emrah.oguz@ege.edu.tr (E.O.); vusali.kasumovi@ege.edu.tr (V.K.); aysen.yaprak.kapkin@ege.edu.tr (A.Y.K.); ahmet.daylan@ege.edu.tr (A.D.); serkan.ertugay@ege.edu.tr (S.E.); cagatay.engin@ege.edu.tr (C.E.); mustafa.ozbaran@ege.edu.tr (M.O.); 2 Department of Cardiology, Faculty of Medicine, Ege University, 35100 Izmir, Turkey; sanem.nalbantgil@ege.edu.tr

**Keywords:** left ventricular assist device, Doppler blood pressure, mean arterial pressure, Bland–Altman analysis, non-pulsatile flow, invasive blood pressure monitoring

## Abstract

**Background:** Continuous-flow left ventricular assist devices (LVADs) generate non-pulsatile circulation, rendering conventional oscillometric blood pressure measurements unreliable. Accurate monitoring is critical to prevent complications including stroke, pump thrombosis, and aortic regurgitation. Doppler-based measurement is widely used as a non-invasive alternative, yet its accuracy relative to invasive arterial pressure remains insufficiently characterized. **Methods:** In this prospective single-centre study, 32 adult continuous-flow LVAD patients underwent simultaneous invasive radial artery and Doppler blood pressure measurements twice daily over three consecutive days (192 paired readings; Day 3: n = 27 due to technical recording issues). Pulsatility was assessed by means of peripheral pulse palpation and transthoracic echocardiography. Spearman’s rho, Wilcoxon signed-rank test, and Bland–Altman analysis were applied. **Results:** Median invasive MAP was 73.0 [IQR 66–80] mmHg and median Doppler pressure was 75.0 [IQR 70–80] mmHg. Doppler measurements demonstrated strong-to-excellent correlation with invasive MAP across all time points (r = 0.78–0.91, *p* < 0.001), with no significant paired differences (all *p* > 0.05). Bland–Altman analysis revealed a bias of −0.35 mmHg with limits of agreement of −9.10 to +8.40 mmHg, within the accepted ±10 mmHg threshold. Correlation with systolic pressure was lower (r = 0.66–0.89, *p* < 0.001), with a positive bias of +13.47 mmHg and wide limits of agreement (+1.28 to +25.67 mmHg), indicating clinically unacceptable agreement. **Conclusions:** Doppler-derived blood pressure may provide a reliable estimate of invasive MAP in continuous-flow LVAD patients, whereas its utility for systolic pressure estimation appears limited. Doppler measurement represents a practical, non-invasive tool for routine MAP monitoring in both inpatient and outpatient settings.

## 1. Introduction

Long-term implantation of left ventricular assist devices (LVADs) has become an established and life-saving therapeutic option for patients with end-stage heart failure. Compared to earlier pulsatile-flow devices, contemporary continuous-flow LVADs, with their smaller size, improved durability, and less invasive implantation techniques, have significantly enhanced survival rates and quality of life. However, despite these technological advances, LVAD recipients remain at considerable risk for complications such as infection, bleeding, thrombosis, and cerebrovascular events [1].

Continuous-flow LVADs generate a non-pulsatile blood flow, leading to significant alterations in physiological hemodynamics. This flow pattern is characterized by increased diastolic blood pressure and reduced pulse pressure, which in turn results in an elevation of mean arterial pressure (MAP). Consequently, conventional blood pressure measurement techniques, including auscultatory and oscillometric methods, lose their reliability due to diminished or absent pulsatility. In particular, oscillometric devices often fail to provide accurate measurements or may not yield any readings in this patient population [2].

Accurate monitoring of blood pressure is of critical importance in LVAD patients, as hypertension represents a major modifiable risk factor for adverse outcomes such as pump thrombosis, stroke, and aortic regurgitation. Due to the limitations of conventional methods, alternative techniques such as Doppler-based blood pressure measurement have been increasingly adopted in clinical practice. Doppler ultrasonography enables detection of blood flow even under low-pulsatility conditions and therefore represents a promising non-invasive approach.

However, despite its widespread clinical use, the accuracy and reliability of Doppler-derived blood pressure measurements in comparison with invasive arterial pressure monitoring remain controversial. In particular, it is unclear whether Doppler measurements more accurately reflect systolic blood pressure or mean arterial pressure in patients with continuous-flow LVADs.

Therefore, the aim of this study was to compare non-invasive Doppler blood pressure measurements with invasive arterial pressure measurements and to evaluate the reliability of Doppler-derived values in patients receiving continuous-flow LVAD support.

## 2. Materials and Methods

This study was conducted at the Cardiovascular Surgery Department of Ege University and was supported by the Ege University Scientific Research Projects Coordination Unit (Project Number: 23685). Ethical approval was obtained from the institutional ethics committee (approval number: 22-2.2/4). Written informed consent was obtained from all participants in accordance with the Declaration of Helsinki, the Personal Data Protection Law No. 6698, and the Regulation on Personal Health Data.

The study included 32 adult patients (≥18 years) who underwent continuous-flow left ventricular assist device (LVAD) implantation for heart failure and had invasive arterial pressure monitoring via a radial artery catheter. Patients with known peripheral arterial disease were excluded from the study.

Blood pressure measurements were performed simultaneously using invasive arterial monitoring and a non-invasive vascular Doppler device in the same extremity.

Invasive arterial pressure was continuously monitored via a radial artery catheter using a bedside monitor (Nihon Kohden Life Scope, Nihon Kohden Corporation, Tokyo, Japan).

For non-invasive measurements, a handheld Doppler device (Hadeco, Inc., Kawasaki, Japan) with an 8 MHz probe was used. The Doppler probe was positioned over the brachial artery at the antecubital fossa, and a standard blood pressure cuff was placed proximally on the ipsilateral arm. The cuff was inflated to occlude arterial flow and gradually deflated. The pressure at which the Doppler signal reappeared was recorded as the Doppler blood pressure. 

Measurements were obtained twice daily for three consecutive days, and invasive arterial pressure values were recorded simultaneously with Doppler measurements, resulting in a total of 192 paired measurements. Five patients did not complete Day 3 measurements due to technical recording issues during data acquisition; therefore, Day 3 analyses were based on 27 patients.

Pulsatility was assessed through simultaneous palpation of the peripheral pulse and evaluation of invasive arterial pressure waveforms during blood pressure measurements. In addition, transthoracic echocardiography was used to assess aortic valve opening patterns, categorized as continuously closed, intermittent opening, or continuously open.

Demographic and clinical data were recorded for all patients, including age, sex, body mass index (BMI), comorbidities, previous surgical history, use of antihypertensive medications, and presence of pulsatile flow. Device type and INTERMACS classification were also documented.

## 3. Statistical Analysis

Statistical analyses were performed using SPSS software (IBM SPSS Statistics for MacOS, Version 30.0: IBM Corp., Armonk, NY, USA). Continuous variables with non-normal distribution are presented as median (interquartile range) [IQR]; normally distributed continuous variables (age and body mass index) are reported as mean ± standard deviation. Categorical variables are presented as frequencies and percentages.

The distribution of data was assessed using the Shapiro–Wilk test, which indicated a non-normal distribution. Therefore, non-parametric statistical methods were used. Because repeated blood pressure measurements were obtained from each participant over the three-day study period, patient-level averaged values were calculated for systolic arterial pressure, invasive mean arterial pressure (MAP), and Doppler blood pressure prior to Bland–Altman analysis. Accordingly, each patient contributed a single averaged observation (n = 32) to the Bland–Altman agreement analysis rather than all repeated measurements being treated as independent observations.

Spearman’s rank correlation coefficient was applied to evaluate the relationship between Doppler blood pressure measurements and invasive arterial pressure measurements (mean and systolic). Correlation strength was interpreted as follows: 0.00–0.19 (very weak), 0.20–0.39 (weak), 0.40–0.59 (moderate), 0.60–0.79 (strong), and ≥0.80 (excellent).

The Wilcoxon signed-rank test was used to compare paired Doppler and invasive arterial pressure measurements, applied separately for mean arterial pressure (MAP) and systolic arterial pressure comparisons.

Agreement between the two measurement methods was further evaluated using Bland–Altman analysis. For each pair of measurements, the difference (reference measurement—Doppler measurement) and the mean of the two methods were calculated. The mean difference (bias), limits of agreement (bias ± 1.96 × standard deviation), and 95% confidence intervals were determined. Bland–Altman plots were constructed to assess the distribution of differences and the presence of systematic bias.

A two-tailed *p*-value < 0.05 was considered statistically significant.

## 4. Results

A total of 32 patients were included in the study. The majority of patients were male (96.9%), with a mean age of 52.3 ± 8.2 years. Dilated cardiomyopathy (DCM) was present in 56.3% of patients, while 43.8% had ischemic cardiomyopathy (ICP). Device distribution was as follows: 6.3% HeartMate II (Abbott Inc., Abbott Park, IL, USA), 84.4% HeartMate 3 (Abbott Inc., Abbott Park, IL, USA), and 9.4% HeartWare (Medtronic, Inc., Minneapolis, MN, USA). The mean body mass index (BMI) was 25.9 kg/m^2^. Hypertension was present in 21.9% of patients, and diabetes mellitus in 40.6%. No patients had peripheral arterial disease, while 3.1% had a history of prior coronary artery bypass surgery. Regarding antihypertensive therapy, 12.5% of patients were not receiving any treatment. According to INTERMACS classification, 9.4% of patients were profile 1, 18.8% profile 2, 34.4% profile 3, 28.1% profile 4, 3.1% profile 5, and 6.3% profile 6 (Table 1).

Postoperative assessment revealed palpable peripheral pulses in 25% of patients. Echocardiographic evaluation demonstrated no aortic valve opening in 34.4% of patients, continuous opening in 28.1%, and intermittent opening in 37.5% (Table 2). Invasive arterial pressure measurements demonstrated a median systolic blood pressure of 88.0 (IQR: 77.0–99.0) mmHg, median diastolic blood pressure of 64.5 (IQR: 54.0–72.0) mmHg, and median mean arterial pressure (MAP) of 73.0 (IQR: 66.0–80.0) mmHg. Median pulse pressure was 23.5 (IQR: 17.0–30.8) mmHg. Median Doppler blood pressure was 75.0 (IQR: 70.0–80.0) mmHg.

Spearman correlation analysis comparing invasive mean arterial pressure (MAP) and Doppler measurements demonstrated strong to excellent correlations across all time points (r = 0.78–0.91, *p* < 0.001) (Table 3). Day 1 and Day 2 analyses included all 32 patients; Day 3 analyses included 27 patients due to technical recording issues in five cases. The highest correlation was observed at Day 1–2 (r = 0.910), followed by Day 2–1 (r = 0.906), Day 2–2 (r = 0.878), Day 3–2 (r = 0.803), Day 3–1 (r = 0.788), and Day 1–1 (r = 0.780). Wilcoxon signed-rank test showed no statistically significant differences between Doppler and invasive MAP measurements at any time point (all *p* > 0.05), indicating good agreement between the two methods (Table 4).

In contrast, when Doppler measurements were compared with invasive systolic arterial pressure, correlations remained strong to excellent but were relatively lower (r = 0.66–0.89, *p* < 0.001) (Table 3). The highest correlation was observed at Day 2–2 (r = 0.894), followed by Day 1–2 (r = 0.801), Day 2–1 (r = 0.800), Day 3–1 (r = 0.721), Day 1–1 (r = 0.689), and Day 3–2 (r = 0.664). However, the Wilcoxon signed-rank test revealed statistically significant differences between Doppler and invasive systolic blood pressure measurements at all time points (all *p* < 0.001), indicating that Doppler measurements are not consistent with systolic arterial pressure (Table 4).

**Figure 1:**
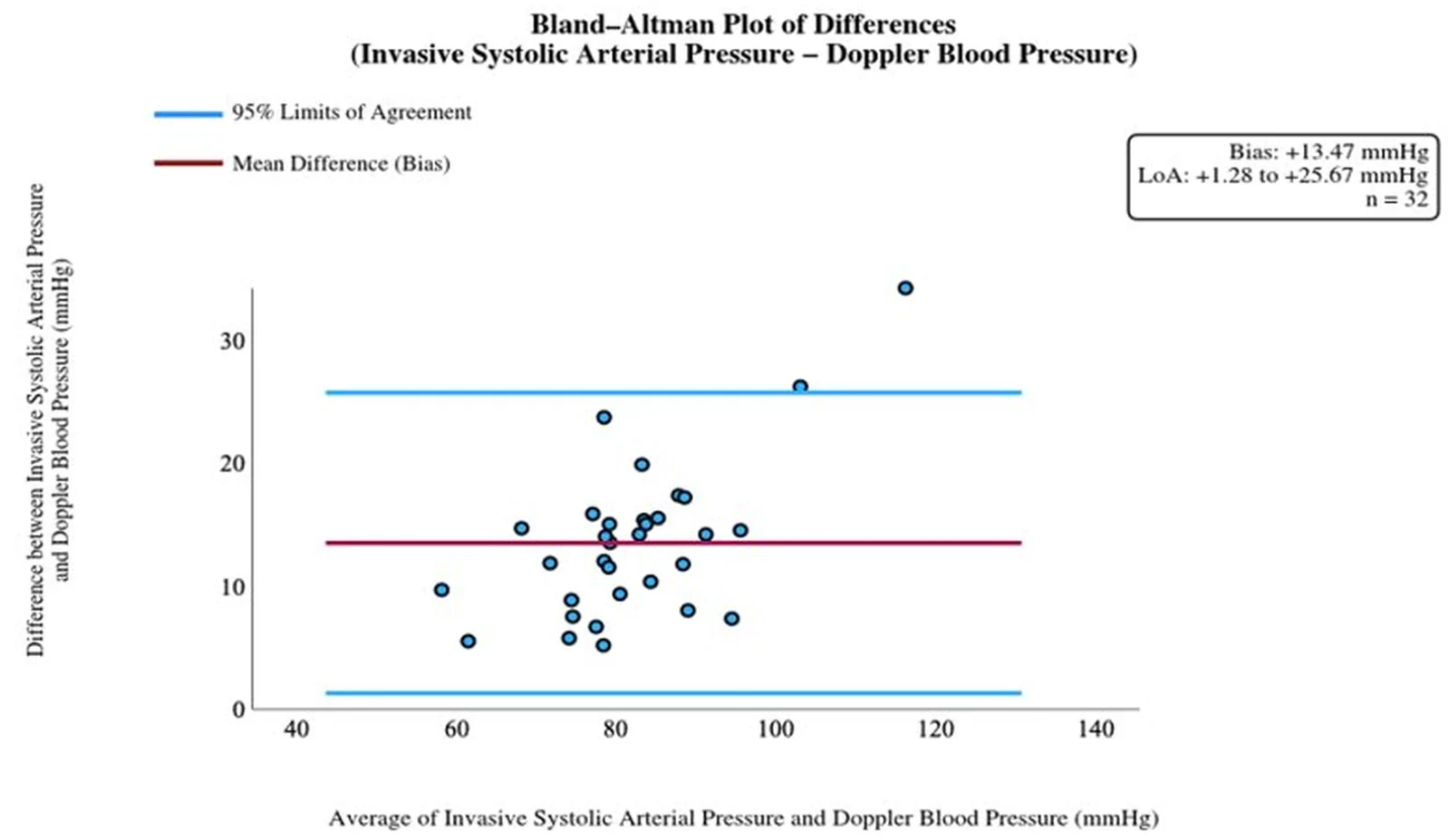
Bland–Altman plot of the difference between systolic arterial pressure and vascular Doppler pressure. The x-axis represents the average of invasive systolic arterial pressure and Doppler blood pressure (mmHg); the y-axis represents the difference between invasive systolic arterial pressure and Doppler blood pressure (mmHg). The solid line indicates the mean bias (+13.47 mmHg); dashed lines indicate the 95% limits of agreement (+1.28 to +25.67 mmHg), indicating clinically unacceptable agreement at the individual level. Each data point represents the patient-level averaged value (n = 32).

Bland–Altman analysis for systolic blood pressure revealed a substantial positive mean bias of +13.47 mmHg, with wide limits of agreement ranging from +1.28 to +25.67 mmHg (Table 5). These findings indicate a systematic overestimation of systolic blood pressure by Doppler measurements and considerable variability at the individual level. Although no proportional bias was detected (*p* = 0.18), the wide limits of agreement suggest poor clinical reliability for estimating systolic blood pressure (Figure 1).

**Figure 2:**
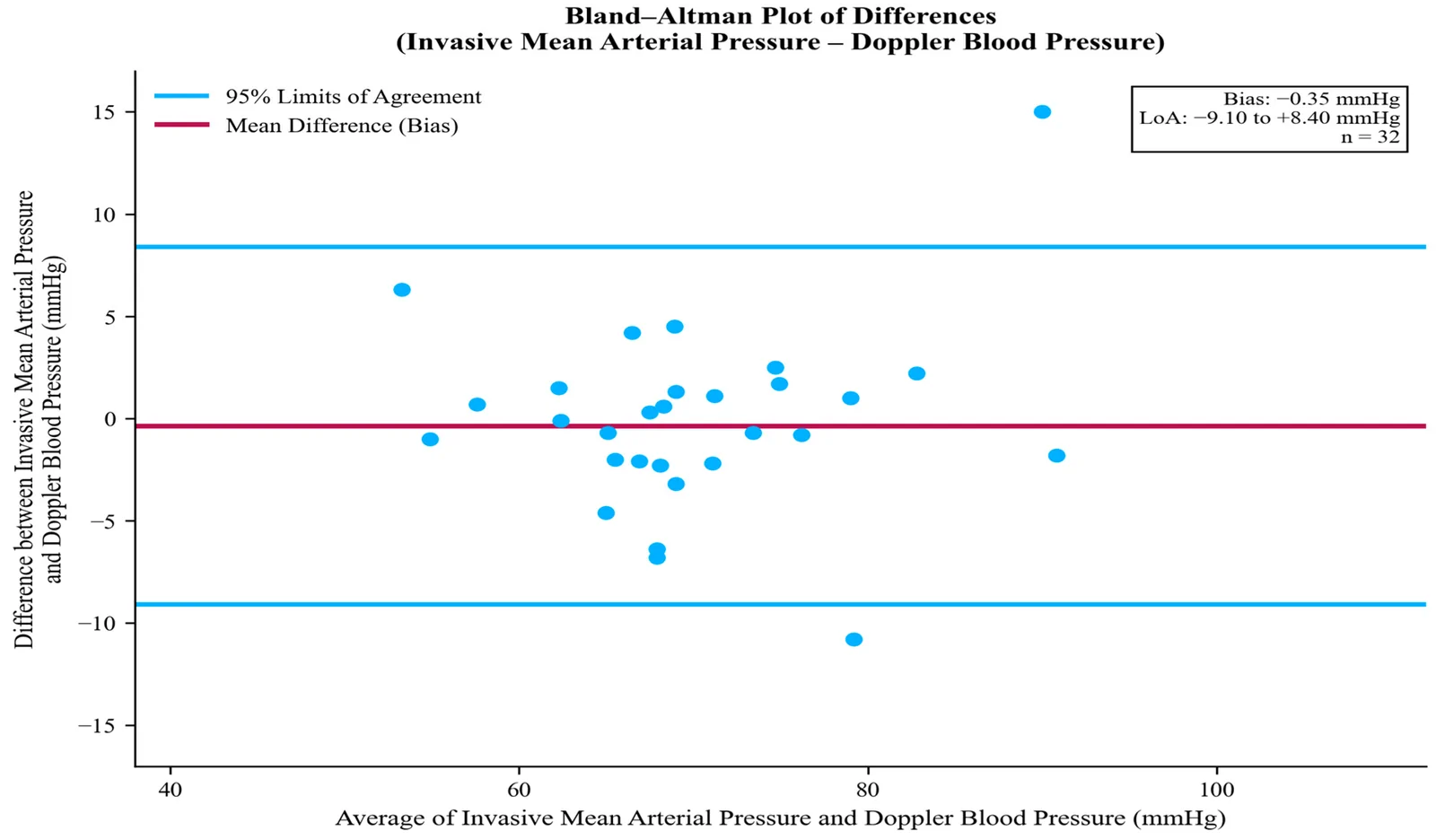
Bland–Altman plot of the differences between mean arterial pressure and vascular Doppler pressure. The x-axis represents the average of invasive mean arterial pressure and Doppler blood pressure (mmHg); the y-axis represents the difference between invasive mean arterial pressure and Doppler blood pressure (mmHg). The solid line indicates the mean bias (−0.35 mmHg); dashed lines indicate the 95% limits of agreement (−9.10 to +8.40 mmHg), falling within the accepted ±10 mmHg threshold, indicating clinically acceptable agreement. Each data point represents the patient-level averaged value (n = 32).

Bland–Altman agreement analyses were performed using patient-level averaged blood pressure values derived from repeated measurements obtained during the study period. Bland–Altman analysis demonstrated a strong agreement between Doppler measurements and invasive MAP. The mean bias was −0.35 mmHg, with limits of agreement ranging from −9.10 to +8.40 mmHg (Table 5). These values fall within the ±10 mmHg range defined by international validation standards, indicating agreement within the conventionally accepted ±10 mmHg range. No significant proportional bias was observed (*p* = 0.42), and the differences were evenly distributed across the measurement range (Figure 2).

## 5. Discussion

Hypertension is a well-established risk factor for both ischemic and hemorrhagic stroke [3]. In patients with left ventricular assist devices (LVADs), blood pressure control is of particular importance, as these devices are preload dependent but highly sensitive to afterload, and Increases in systemic blood pressure may reduce pump flow and impair left ventricular unloading [4,5]. Poorly controlled hypertension has been associated with increased risks of hemorrhagic stroke, aortic regurgitation, and thromboembolic events in LVAD patients [4,6]. In our cohort, hypertension was present in 21.9% of patients, and the majority were receiving antihypertensive therapy (87.5%), highlighting the clinical burden of blood pressure management in this population and the need for reliable non-invasive monitoring methods.

Although hypertension has traditionally been emphasized as the primary blood pressure-related complication in LVAD patients, recent evidence demonstrates that chronically low blood pressure also carries significant risk. Cowger et al. analyzed data from the INTERMACS registry and found that patients with chronically low MAP (≤75 mmHg), Doppler-derived MAP ≤ 80 mmHg, or systolic blood pressure (SBP) < 90 mmHg had 35–42% higher adjusted hazards of death compared with patients with normal or high blood pressure (*p* ≤ 0.0001) [7]. Notably, the median invasive MAP was 73.0 mmHg, the median Doppler-derived pressure was 75.0 mmHg, and the median systolic blood pressure was 88.0 mmHg—all of which fall within the high-risk thresholds defined by Cowger et al., even in the context of ongoing antihypertensive therapy. The coexistence of hypertension and hypotension risk in the same population highlights that both extremes must be carefully monitored, making precise and reliable hemodynamic monitoring essential for safe clinical management.

The HVAD: ENDURANCE Supplemental Trial demonstrated that implementing a blood pressure control protocol aimed at reducing MAP was associated with lower rates of adverse events such as stroke and aortic regurgitation [8]. Expert consensus recommends maintaining blood pressure within defined target ranges during continuous-flow LVAD support. For non-pulsatile patients, a mean arterial pressure (MAP) below 80 mmHg is recommended, while those with preserved pulsatility should maintain a systolic blood pressure below 130 mmHg and a diastolic blood pressure below 85 mmHg; more recently, a MAP target of 75–90 mmHg has been proposed regardless of pulsatility status [4,9]. In our cohort, 75% of patients had non-palpable peripheral pulses, and the median Doppler-derived pressure was 75.0 mmHg, which approaches the lower boundary of the recommended target range.

In addition to cerebrovascular and valvular complications, systemic hypertension has also been linked to device-specific adverse events such as pump thrombosis. Najjar et al. analyzed pump thrombus events in 382 patients who underwent centrifugal continuous-flow LVAD implantation as a bridge to transplant in the HeartWare ADVANCE trial. Their multivariable analysis demonstrated that a mean arterial pressure (MAP) greater than 90 mmHg was significantly associated with an increased risk of pump thrombosis [10]. Taken together, the risks associated with both extremes of blood pressure, the importance of maintaining MAP within defined target ranges, and the limitations of conventional oscillometric methods in non-pulsatile LVAD patients collectively highlight the need for an accurate non-invasive alternative. In this context, we prospectively evaluated the accuracy of Doppler-derived blood pressure measurements against invasive arterial pressure through repeated simultaneous measurements obtained twice daily over three consecutive days in the early postoperative period.

Doppler measurements demonstrated strong to excellent correlation with invasive MAP across all time points (r = 0.78–0.91, *p* < 0.001), with no significant differences observed in paired comparisons. Bland–Altman analysis revealed minimal bias (−0.35 mmHg) and limits of agreement within the conventionally accepted ±10 mmHg range (−9.10 to +8.40 mmHg), supporting the potential clinical utility of Doppler measurements for estimating MAP. In contrast, although Doppler measurements showed strong to excellent correlation with invasive systolic arterial pressure (r = 0.66–0.89, *p* < 0.001), statistically significant differences were observed at all time points, and Bland–Altman analysis revealed a substantial positive bias of +13.47 mmHg with wide limits of agreement (+1.28 to +25.67 mmHg), indicating clinically unacceptable agreement at the individual level. These findings are consistent with previous reports: Li et al. similarly demonstrated that Doppler-derived pressure correlates closely with invasive MAP and is not significantly affected by arterial pulsatility [11], and Rangasamy et al. reported that Doppler measurements better reflect MAP in patients without palpable pulse, whereas correlation with systolic pressure is more variable [12]. While Li et al. reported a mean error of 2.4 mmHg without formal agreement analysis, and Rangasamy et al. relied on pulse palpability to guide Doppler interpretation, neither study performed Bland–Altman analysis to quantify limits of agreement. The present study extends these findings by demonstrating clinically acceptable agreement for MAP, regardless of pulse palpability. Furthermore, our cohort was predominantly composed of HeartMate 3 patients (84.4%), providing contemporary validity in a device type underrepresented in prior studies. These findings suggest that Doppler-derived values may reliably reflect MAP in continuous-flow LVAD patients; however, even relatively small deviations in blood pressure may have clinical relevance in this population and should therefore be interpreted within the broader clinical context.

Importantly, our results confirm that Doppler blood pressure measurements in continuous-flow LVAD patients more closely reflect MAP rather than systolic pressure. This distinction is clinically relevant, as MAP is the primary parameter recommended for blood pressure management in this population. From a practical perspective, Doppler measurement is a simple, non-invasive, and accessible method that can be applied in both inpatient and outpatient settings. It can be performed not only by LVAD coordinators but also by trained healthcare personnel and caregivers, facilitating its widespread use in routine clinical practice [13].

Although the number of studies on this topic remains limited, our findings contribute to the growing body of evidence supporting Doppler measurement as a reliable and clinically applicable method for blood pressure assessment in patients with continuous-flow LVADs.

## 6. Limitations

This study has several limitations. First, the relatively small sample size (n = 32) and single-center design may limit the generalizability of the findings. A formal power calculation was not performed prior to the study, which should be considered when interpreting the results.

Second, although measurements were performed over three consecutive days, the short follow-up period may not fully reflect long-term hemodynamic variability in LVAD patients.

Third, five patients did not complete Day 3 measurements due to technical recording issues during data acquisition, resulting in incomplete follow-up data for those participants. Although this represents a minority of the cohort (15.6%), it may have introduced selection bias in the Day 3 analyses and should be considered when interpreting the three-day longitudinal findings. 

Fourth, Doppler measurements were performed by multiple LVAD coordinators, which may have introduced inter-operator variability. 

Fifth, the inclusion of different LVAD device types (HeartMate II, HeartMate 3, and HVAD) may have influenced hemodynamic characteristics and measurement accuracy, potentially affecting the comparability of results.

Sixth, the study population was almost exclusively male (96.9%), which limits the generalizability of the findings to female LVAD patients. Future studies with larger and more sex-balanced cohorts are warranted.

Seventh, invasive arterial pressure was recorded immediately before cuff inflation and immediately after cuff deflation, and only measurements in which both values were concordant were included in the analysis. However, the possibility that cuff compression during the inflation period may have transiently influenced the radial artery pressure cannot be entirely excluded. This represents an inherent methodological limitation of simultaneous ipsilateral Doppler and invasive pressure measurement.

Finally, repeated measurements were obtained from the same participants during the study period. Although Bland–Altman analyses were performed using patient-level averaged values, the serial correlation structure between repeated measurement sessions was not formally modeled. Therefore, the time-point-specific analyses should be interpreted as exploratory descriptive comparisons.

## 7. Conclusions

In patients with continuous-flow left ventricular assist devices, Doppler-derived blood pressure may provide a reliable estimate of invasive mean arterial pressure, whereas its utility for systolic blood pressure estimation appears limited. 

Therefore, Doppler-derived blood pressure may be considered a practical and reliable non-invasive method for routine blood pressure monitoring in continuous-flow LVAD patients, particularly for the assessment and management of mean arterial pressure. These findings support the routine use of Doppler-derived MAP in the perioperative and long-term management of LVAD patients.

## Figures and Tables

**Table 1:** Baseline Demographic and Clinical Characteristics.

Characteristic	Mean ± SD or n (%)
Demographics	
Age, years	52.3 ± 8.2
Male sex, n (%)	31 (96.9)
Body mass index, kg/m^2^	25.9 ± 4.2
Cardiomyopathy etiology	
Dilated cardiomyopathy, n (%)	18 (56.3)
Ischemic cardiomyopathy, n (%)	14 (43.8)
Comorbidities	
Hypertension, n (%)	7 (21.9)
Diabetes mellitus, n (%)	13 (40.6)
Prior coronary bypass surgery, n (%)	1 (3.1)
Device type	
HeartMate 3, n (%)	27 (84.4)
HVAD, n (%)	3 (9.4)
HeartMate II, n (%)	2 (6.3)
INTERMACS profile	
Profile 1 (Critical cardiogenic shock), n (%)	3 (9.4)
Profile 2 (Progressive decline), n (%)	6 (18.8)
Profile 3 (Stable but inotrope dependent), n (%)	11 (34.4)
Profile 4 (Resting symptoms), n (%)	9 (28.1)
Profile 5 (Exertion intolerant), n (%)	1 (3.1)
Profile 6 (Exertion limited), n (%)	2 (6.3)
Antihypertensive therapy	
None, n (%)	4 (12.5)
Single agent, n (%)	16 (50.0)
Dual therapy, n (%)	10 (31.3)
Triple therapy, n (%)	2 (6.3)

INTERMACS: Interagency Registry for Mechanically Assisted Circulatory Support. Continuous variables are presented as mean ± SD; categorical variables as n (%).

**Table 2:** Hemodynamic Parameters and Pulsatility Assessment.

Parameter	Median [IQR] or n (%)
**Invasive arterial pressure, mmHg**	
Systolic blood pressure, mmHg	88.0 [77.0–99.0]
Diastolic blood pressure, mmHg	64.5 [54.0–72.0]
Mean arterial pressure, mmHg	73.0 [66.0–80.0]
**Doppler blood pressure, mmHg**	
Mean Doppler pressure	75.0 [70.0–80.0]
**Pulse palpation, n (%)**	
Palpable	8 (25.0)
Non-palpable	24 (75.0)
**Aortic valve opening, n (%)**	
Continuously closed	11 (34.4)
Intermittent opening	12 (37.5)
Continuously open	9 (28.1)

Values are presented as Median [IQR] for continuous variables and n (%) for categorical variables. Invasive arterial pressure was monitored continuously via radial artery catheter. Doppler blood pressure was measured using an 8 MHz handheld Doppler probe positioned over the brachial artery. Pulsatility was assessed by means of peripheral pulse palpation and transthoracic echocardiography. MAP: mean arterial pressure; IQR: interquartile range. Individual patient-level measurement data, including daily invasive arterial and Doppler blood pressure values, pulse palpation results, and aortic valve opening patterns, are provided in Supplementary Table S1.

**Table 3:** Spearman Correlation Between Doppler Blood Pressure and Invasive Arterial Pressure.

			Doppler vs. MAP			Doppler vs. Systolic BP
Time	Spearman’s Rho (r)	*p*-Value	Strength	Spearman’s Rho (r)	*p*-Value	Strength
Day 1–1	0.780	<0.001	Strong	0.689	<0.001	Strong
Day 1–2	0.910	<0.001	Excellent	0.801	<0.001	Excellent
Day 2–1	0.906	<0.001	Excellent	0.800	<0.001	Excellent
Day 2–2	0.878	<0.001	Excellent	0.894	<0.001	Excellent
Day 3–1	0.788	<0.001	Strong	0.721	<0.001	Strong
Day 3–2	0.803	<0.001	Excellent	0.664	<0.001	Strong

Spearman’s rho (r) was used to assess the correlation between Doppler blood pressure measurements and invasive arterial pressure at each time point over three consecutive days (two measurements per day). Doppler measurements demonstrated strong to excellent correlation with invasive mean arterial pressure (MAP) across all time points (r = 0.78–0.91, *p* < 0.001), with the highest correlation observed at Day 1–2 (r = 0.910). Correlation with invasive systolic arterial pressure was also strong to excellent but consistently lower than MAP correlations across all time points (r = 0.66–0.89, *p* < 0.001), with the highest correlation at Day 2–2 (r = 0.894). Correlation strength was classified according to established thresholds: 0.00–0.19 = very weak; 0.20–0.39 = weak; 0.40–0.59 = moderate; 0.60–0.79 = strong; ≥0.80 = excellent. All *p* values < 0.001. Full Spearman correlation outputs for mean arterial pressure and systolic arterial pressure are provided in Supplementary Tables S2 and S5, respectively.

**Table 4:** Wilcoxon Signed-Rank Test: Doppler vs. Invasive Arterial Pressure.

			Doppler vs. MAP			Doppler vs. Systolic BP
Time	Z	*p*-Value	Interpretation	Z	*p*-Value	Interpretation
Day 1–1	−0.762	0.446	NS	−4.863	<0.001	Significant
Day 1–2	−0.065	0.948	NS	−4.923	<0.001	Significant
Day 2–1	−1.139	0.255	NS	−4.597	<0.001	Significant
Day 2–2	−1.550	0.121	NS	−4.864	<0.001	Significant
Day 3–1	−0.561	0.575	NS	−4.371	<0.001	Significant
Day 3–2	−0.213	0.831	NS	−4.122	<0.001	Significant

The Wilcoxon signed-rank test was applied to assess systematic differences between paired Doppler blood pressure and invasive arterial pressure measurements obtained simultaneously at each of six time points over three consecutive days (two measurements per day, n = 32 per time point for Days 1–2 and n = 27 for Day 3). Z denotes the Wilcoxon signed-rank test statistic, a standardized value derived from the sum of positive and negative signed ranks; larger absolute Z values indicate greater systematic difference between the paired measurements. For Doppler vs. invasive MAP comparisons, Z values were small in absolute magnitude (range: −1.550 to −0.065) and all *p* values exceeded 0.05 (range: 0.121–0.948), indicating no statistically significant difference between Doppler and invasive MAP measurements at any time point. For Doppler vs. invasive systolic arterial pressure comparisons, Z values were large in absolute magnitude (range: −4.923 to −4.122), with all *p* values < 0.001, indicating statistically significant and systematic overestimation of systolic blood pressure by Doppler measurements at all time points. Z: Wilcoxon signed-rank test statistic; NS: not significant (*p* > 0.05); MAP: mean arterial pressure. Full Wilcoxon signed-rank test outputs for mean arterial pressure and systolic arterial pressure are provided in Supplementary Tables S3 and S4, respectively.

**Table 5:** Bland–Altman Analysis: Agreement Between Doppler and Invasive Arterial Pressure.

		Doppler vs. Systolic BP		Doppler vs. MAP
Parameter	Value	95% CI	Value	95% CI
Bias (mean difference), mmHg	13.47	11.23–15.72	−0.35	−1.96–1.26
Lower LoA, mmHg	1.28	−2.60–5.15	−9.10	−11.88–−6.32
Upper LoA, mmHg	25.67	21.80–29.55	8.40	5.62–11.18

Bland–Altman analysis was performed to assess agreement between Doppler blood pressure measurements and invasive arterial pressure. For each pair of simultaneous measurements, the difference (invasive − Doppler) and the mean of the two values were calculated. Bias represents the mean difference between methods; limits of agreement (LoA) were calculated as bias ± 1.96 × SD and define the interval within which 95% of individual differences are expected to fall. For Doppler vs. MAP, bias was −0.35 mmHg with LoA of −9.10 to +8.40 mmHg, falling within the internationally accepted ±10 mmHg threshold, indicating agreement within the conventionally accepted ±10 mmHg range. No proportional bias was detected (*p* = 0.42). For Doppler vs. systolic arterial pressure, bias was +13.47 mmHg with wide LoA of +1.28 to +25.67 mmHg, indicating that Doppler measurements systematically overestimate systolic blood pressure relative to invasive measurement and clinically unacceptable agreement at the individual level. No proportional bias was detected (*p* = 0.18). Bias: mean difference between invasive and Doppler measurements; LoA: limits of agreement (bias ± 1.96 × SD); CI: confidence interval; MAP: mean arterial pressure; SD: standard deviation.

## Data Availability

The original contributions presented in this study are included in the article/Supplementary Materials. Further inquiries can be directed to the corresponding author.

## Supplementary Materials

The following supporting information can be downloaded at https://www.mdpi.com/article/10.3390/jcdd13060276/s1. Table S1: Daily invasive arterial–Doppler pressure measurements and pulse palpation with aortic valve opening; Table S2: Spearman correlation analysis—Doppler device blood pressure measurement with mean arterial pressure; Table S3: Wilcoxon Signed Rank Test—Doppler measurement pressure with mean arterial pressure; Table S4: Wilcoxon Signed Rank Test—Comparison of systolic blood pressure and Doppler device blood pressure measurement; Table S5: Comparison of systolic arterial pressure and blood pressure measurement of the Doppler device—Spearman correlation analysis.
