# Supplementary material for: Clinical Evaluation of Doppler Blood Pressure Measurement in Continuous-Flow LVAD Patients: Implications for Postoperative Management
Source: J Cardiovasc Dev Dis. 2026 Jun 18;13(6):276. doi: 10.3390/jcdd13060276 (PMC13302551; doi:10.3390/jcdd13060276)
Supplement: Supplementary file 1 [file jcdd-13-00276-s001.zip › jcdd-4305744-supplementary.pdf]

Table S1: Daily invasive arterial-Doppler pressure measurements and pulse palpation with aortic valve opening

Patient number	*1-1 Day Invasive arteryel pressure mm/hg*	*1-2 Day Invasive arteryel Pressure*	*1-1 Day Vascular doppler Blood pressure*	*1-2 Day Vascular doppler Blood pressure*	*2-1 Day Invasive arteryel pressure mm/hg*	*2-2 Day Invasive arteryel Pressure*	*2-1 Day Vascular doppler Blood pressure*	*2-2 Day Vascular doppler Blood pressure*	*3-1 Day Invasive arteryel pressure mm/hg*	*3-2 Day Invasive arteryel Pressure*	*3-1 Day Vascular doppler Blood pressure*	*3-2 Day Vascular doppler Blood pressure*	*Palpability*	*Aort valve Opening*
1	114/29 73	116/30 77	70	75	70/50 65	80/51 63	80	70	70/68 69	66/48 60	70	70	None	None
2	101/77 91	92/66 83	80	80	69/49 54	72/55 64	65	70	75/48 61	77/52 65	80	80	None	Per 3 beat
3	88/70 78	82/64 71	70	70	98/72 80	76/55 68	75	70	82/74 76	94/77 84	75	70	None	None
4	82/59 69	80/53 65	70	65	99/79 80	95/75 87	85	85	96/74 82	95/87 79	75	75	Palpable	In every beat
5	75/65 69	74/63 68	70	70	69/23 64	70/24 69	70	70	92/83 73	90/79 69	75	70	None	In every Beat
6	82/74 70	100/67 87	70	85	88/69 88	107/80	85	90					None	Per 2-3 Beat
7	110/80 90	112/78 92	100	110	89/57 62	93/67 76	70	75	103/86 93	82/59 67	95	95	Palpable	In every Beat
8	138/77 94	119/79 91	90	110	120/80 92	147/82 93	100	95	144/102 122	132/74 92	110	90	None	None
9	102/56 75	109/96 102	75	90	108/98 102	101/68 85	90	85	84/62 73	86/65 75	85	80	None	Per 2-3 Beat
10	76/52 65	80/53 68	70	75	92/69 78	91/65 76	80	80	85/62 74	87/59 72	75	75	None	None
11	80/65 72	70/47 62	70	65	83/67 73	80/63 70	75	70	77/61 71	83/58 68	70	70	None	Per 5-6 Beat
12	95/70 85	96/74 90	90	90	89/68 79	92/71 70	70	80	81/68 75	84/71 72	75	70	None	Per 1-2 Beat
13	108/69 92	97/73 88	90	85	97/74 87	112/59 89	85	90	103/81 93	100/84 94	90	90	Palpable	In every beat
14	91/52 66	90/80 85	65	70	78/52 64	74/32 60	65	70	90/64 79	91/69 78	80	80	Palpable	In every Beat
15	89/73 78	99/67 78	75	70	85/70 72	87/71 73	70	70	78/67 68	72/65 71	60	70	None	Per 3 beat
16	95/50 65	90/50 72	80	75	85/48 74	95/50 65	70	80					None	In every Beat
17	65/52 55	65/50 53	60	60	93/60 78	95/67 82	80	80	95/62 78	94/59 77	75	80	None	Per 2-3 Beat
18	120/89 101	119/89 109	90	90	110/84	115/87	90	90	115/82	118/79	90	90	Palpable	In every beat
19	87/86 86	89/70 85	75	75	91/70 83	90/65 78	85	80	112/68 78	110/65 80	80	80	None	Per 2-3 Beat
20	76/54 62	78/57 64	70	70	80/63 76	81/62 73	80	80	110/54 79	115/61 81	75	80	Palpable	None
21	92/60 70	95/67 82	80	85	91/58 68	94/65 81	70	75					None	None
22	84/65 72	70/67 55	65	55	71/53 64	86/62 77	65	70	75/55 66	80/60 70	70	70	None	None
23	86/65 77	81/59 72	80	70	70/52 62	82/60 71	65	75	86/75 78	80/64 72	80	75	None	None
24	87/63 72	92/70 78	70	75	86/75 79	72/58 65	80	70	87/66 74	85/60 73	75	70	Palpable	In every Beat
25	74/56 67	75/57 68	70	70	78/52 60	81/66 72	70	75					None	None
26	51/46 69	71/31 49	40	50	60/51 57	64/51 61	50	60	67/50 62	65/51 60	60	60	None	Per 2-3 beat
27	70/59 71	69/57 69	70	70	102/76 89	105 /78 93	100	90	105/78 95	107/81 98	90	90	Palpable	None
28	99/58 72	100/60 78	70	80	83/67 76	90/72 76	70	80	91/49 71	96/54 75	70	70	None	None
29	76/50 63	75/51 62	60	60	75/51 61	76/52 60	60	60	76/51 62	75/50 61	65	60	None	In 2 beat
30	92/62 77	87/54 70	80	75	102/76 88	101/72 84	85	80	101/75 88	100/60 80	80	80	Palpable	In every Beat
31	73/55 66	60/49 52	60	55	62/49 55	62/48 58	60	60					None	Per 2-3 Beat
32	95/65 65	100/68 68	60	65	100/65 68	100/68 72	70	75	77/55 65	70/52 61	70	60	None	Per 2-3 beat

Table S2: Spearmen correlation analysis – Doppler device blood pressure measurement with mean arterial pressure

			Day_1.1 Invasive arter mean pressure	Day_1.2 Invasive arter mean pressure	Day _1.1 Vascular Doppler Blood pressure	Day _1.2 Vascular Doppler Blood pressure	Day _2.1 Invasive arter mean pressure	Day _2.2 Invasive arter mean pressure	Day _2.1 Vascular Doppler Blood pressure	Day 2.2 Vascular Doppler Blood pressure	Day _3.1 Invasive arter mean pressure	Day _3.2 Invasive arter mean pressure	Day _3.1 Vascular Doppler Blood pressure	Day_3.2 Vascular Doppler Blood pressure	Age	BMI	İNTERMACS
Spearman's rho	Day_1.1 Invasive arter mean pressure	Correlation Coefficient	1,000	.735**	.780**	.660**	.359*	.397*	0,345	0,288	0,354	0,315	.572**	.475*	0,100	0,278	0,125
		Sig. (2-tailed)		0,000	0,000	0,000	0,044	0,025	0,053	0,110	0,070	0,109	0,002	0,012	0,585	0,124	0,496
		N	32	32	32	32	32	32	32	32	27	27	27	27	32	32	32
	Day_1.2 Invasive arter mean pressure	Correlation Coefficient	.735**	1,000	.769**	.910**	.442*	.412*	.402*	.480**	.410*	0,340	.660**	.537**	0,116	.412*	0,173
		Sig. (2-tailed)	0,000		0,000	0,000	0,011	0,019	0,023	0,005	0,034	0,083	0,000	0,004	0,529	0,019	0,343
		N	32	32	32	32	32	32	32	32	27	27	27	27	32	32	32
	Day _1.1 Vascular Doppler Blood pressure	Correlation Coefficient	.780**	.769**	1,000	.829**	.369*	.467**	.382*	.504**	.517**	0,356	.683**	.614**	0,285	0,341	0,101
		Sig. (2-tailed)	0,000	0,000		0,000	0,038	0,007	0,031	0,003	0,006	0,068	0,000	0,001	0,114	0,056	0,583
		N	32	32	32	32	32	32	32	32	27	27	27	27	32	32	32
	Day _1.2 Vascular Doppler Blood pressure	Correlation Coefficient	.660**	.910**	.829**	1,000	.475**	.478**	.486**	.575**	.431*	0,324	.667**	.573**	.359*	0,346	0,075
		Sig. (2-tailed)	0,000	0,000	0,000		0,006	0,006	0,005	0,001	0,025	0,099	0,000	0,002	0,044	0,053	0,685
		N	32	32	32	32	32	32	32	32	27	27	27	27	32	32	32
	Day _2.1 Invasive arter mean pressure	Correlation Coefficient	.359*	.442*	.369*	.475**	1,000	.783**	.906**	.811**	.638**	.806**	.497**	.473*	0,000	0,245	0,019
		Sig. (2-tailed)	0,044	0,011	0,038	0,006		0,000	0,000	0,000	0,000	0,000	0,008	0,013	0,999	0,177	0,918
		N	32	32	32	32	32	32	32	32	27	27	27	27	32	32	32
	Day _2.2 Invasive arter mean pressure	Correlation Coefficient	.397*	.412*	.467**	.478**	.783**	1,000	.783**	.878**	.665**	.684**	.567**	.634**	0,025	0,061	0,132
		Sig. (2-tailed)	0,025	0,019	0,007	0,006	0,000		0,000	0,000	0,000	0,000	0,002	0,000	0,893	0,742	0,472
		N	32	32	32	32	32	32	32	32	27	27	27	27	32	32	32
	Day _2.1 Vascular Doppler Blood pressure	Correlation Coefficient	0,345	.402*	.382*	.486**	.906**	.783**	1,000	.800**	.673**	.722**	.563**	.602**	0,114	0,234	-0,052
		Sig. (2-tailed)	0,053	0,023	0,031	0,005	0,000	0,000		0,000	0,000	0,000	0,002	0,001	0,534	0,198	0,776
		N	32	32	32	32	32	32	32	32	27	27	27	27	32	32	32
	Day 2.2 Vascular Doppler Blood pressure	Correlation Coefficient	0,288	.480**	.504**	.575**	.811**	.878**	.800**	1,000	.742**	.748**	.667**	.700**	0,026	0,135	0,058
		Sig. (2-tailed)	0,110	0,005	0,003	0,001	0,000	0,000	0,000		0,000	0,000	0,000	0,000	0,886	0,463	0,752
		N	32	32	32	32	32	32	32	32	27	27	27	27	32	32	32
	Day _3.1 Invasive arter mean pressure	Correlation Coefficient	0,354	.410*	.517**	.431*	.638**	.665**	.673**	.742**	1,000	.815**	.788**	.803**	0,145	.418*	0,266
		Sig. (2-tailed)	0,070	0,034	0,006	0,025	0,000	0,000	0,000	0,000		0,000	0,000	0,000	0,470	0,030	0,180
		N	27	27	27	27	27	27	27	27	27	27	27	27	27	27	27
	Day _3.2 Invasive arter mean pressure	Correlation Coefficient	0,315	0,340	0,356	0,324	.806**	.684**	.722**	.748**	.815**	1,000	.626**	.655**	-0,096	.417*	0,319
		Sig. (2-tailed)	0,109	0,083	0,068	0,099	0,000	0,000	0,000	0,000	0,000		0,000	0,000	0,634	0,030	0,105
		N	27	27	27	27	27	27	27	27	27	27	27	27	27	27	27
	Day _3.1 Vascular Doppler Blood pressure	Correlation Coefficient	.572**	.660**	.683**	.667**	.497**	.567**	.563**	.667**	.788**	.626**	1,000	.908**	0,169	.383*	0,109
		Sig. (2-tailed)	0,002	0,000	0,000	0,000	0,008	0,002	0,002	0,000	0,000	0,000		0,000	0,400	0,049	0,587
		N	27	27	27	27	27	27	27	27	27	27	27	27	27	27	27
	Day _3.2 Vascular Doppler Blood pressure	Correlation Coefficient	.475*	.537**	.614**	.573**	.473*	.634**	.602**	.700**	.803**	.655**	.908**	1,000	0,113	0,338	0,233
		Sig. (2-tailed)	0,012	0,004	0,001	0,002	0,013	0,000	0,001	0,000	0,000	0,000	0,000		0,575	0,085	0,242
		N	27	27	27	27	27	27	27	27	27	27	27	27	27	27	27
	Age	Correlation Coefficient	0,100	0,116	0,285	.359*	0,000	0,025	0,114	0,026	0,145	-0,096	0,169	0,113	1,000	-0,001	-0,062
		Sig. (2-tailed)	0,585	0,529	0,114	0,044	0,999	0,893	0,534	0,886	0,470	0,634	0,400	0,575		0,997	0,737
		N	32	32	32	32	32	32	32	32	27	27	27	27	32	32	32
	BMI	Correlation Coefficient	0,278	.412*	0,341	0,346	0,245	0,061	0,234	0,135	.418*	.417*	.383*	0,338	-0,001	1,000	0,263
		Sig. (2-tailed)	0,124	0,019	0,056	0,053	0,177	0,742	0,198	0,463	0,030	0,030	0,049	0,085	0,997		0,146
		N	32	32	32	32	32	32	32	32	27	27	27	27	32	32	32
	INTERMACS	Correlation Coefficient	0,125	0,173	0,101	0,075	0,019	0,132	-0,052	0,058	0,266	0,319	0,109	0,233	-0,062	0,263	1,000
		Sig. (2-tailed)	0,496	0,343	0,583	0,685	0,918	0,472	0,776	0,752	0,180	0,105	0,587	0,242	0,737	0,146	
		N	32	32	32	32	32	32	32	32	27	27	27	27	32	32	32

Table S3: Wilcoxon Signed Rank Test - Doppler measurement pressure with mean arterial pressure

Test Statistics[a]

	Day_1.1 Vascular Blood pressure- Day_1.1 Invasive arterial mean pressure	Day_1.2 Vascular Blood pressure- Day_1.2 Invasive arterial mean pressure	Day_2.1 Vascular Blood pressure- Day_2.1 Invasive arterial mean pressure	Day_2.2 Vascular Blood pressure- Day_2.2 Invasive arterial mean pressure	Day_3.1 Vascular Blood pressure- Day_3.1 Invasive arterial mean pressure	Day_3.2 Vascular Blood pressure- Day_3.2 Invasive arterial mean pressure
Z	-.762[b]	-.065[c]	-1.139[c]	-1.550[c]	-.561[b]	-.213[b]
Asymp. Sig. (2-tailed)	0,446	0,948	0,255	0,121	0,575	0,831

Table S4: Wilcoxon Signed Rank Test – Comparison of systolic blood pressure and Doppler device blood pressure measurement

Test Statistics[a]

	DAY.1_1 VASCULAR DOPPLER BLOOD PRESSURE- DAY.1_1 INVASIVE ARTERYEL SYSTOLİC PRESSURE	DAY.1_2 VASCULAR DOPPLER BLOOD PRESSURE - DAY.1_2 INVASIVE ARTERYEL SYSTOLİC PRESSURE	DAY.2_1 - VASCULAR DOPPLER BLOOD PRESSURE DAY.2_1 INVASIVE ARTERYEL SYSTOLİC PRESSURE	DAY.2_2 - VASCULAR DOPPLER BLOOD PRESSURE DAY.2_2 INVASIVE ARTERYEL SYSTOLİC PRESSURE	DAY.3_1 - VASCULAR DOPPLER BLOOD PRESSURE DAY.3_1 INVASIVE ARTERYEL SYSTOLİC PRESSURE	DAY.3_2 - VASCULAR DOPPLER BLOOD PRESSURE DAY.3_2 INVASIVE ARTERYEL SYSTOLİC PRESSURE
Z	-4.863[b]	-4.923[b]	-4.597[b]	-4.864[b]	-4.371[b]	-4.122[b]
Asymp. Sig. (2-tailed)	0,000	0,000	0,000	0,000	0,000	0,000

Table S5: Comparison of systolic arterial pressure and blood pressure measurement of the Doppler device - Spearmen correlation analysis

Correlations

			Day.1_1 Invasive systolic Arteriyal Pressure	Day.1_2 Invasive systolic Arteriyal Pressure	Day.1_1 Vascular doppler Blood Pressure	Day.1_2 Vascular doppler Blood Pressure	Day.2_1 Invasive systolic Arteriyal Pressure	Day.2_2 Invasive systolic Arteriyal Pressure	Day.2_1 Vascular doppler Blood Pressure	Day.2_2 Vascular doppler Blood Pressure	Day.3_1 Invasive systolic Arteriyal Pressure	Day.3_2 Invasive systolic Arteriyal Pressure	Day.3_1 Vascular doppler Blood Pressure	Day.3_2 Vascular doppler Blood Pressure
Spearman's rho	Day.1_1 Invasive systolic Arteriyal Pressure	Correlation Coefficient	1,000	.893**	.689**	.756**	.368*	.418*	0,253	0,309	0,149	0,097	.434*	0,350
		Sig. (2-tailed)		0,000	0,000	0,000	0,038	0,017	0,163	0,086	0,457	0,631	0,024	0,073
		N	32	32	32	32	32	32	32	32	27	27	27	27
	Day.1_2 Invasive systolic Arteriyal Pressure	Correlation Coefficient	.893**	1,000	.596**	.801**	.389*	.462**	0,328	.377*	0,159	0,044	0,330	0,242
		Sig. (2-tailed)	0,000		0,000	0,000	0,028	0,008	0,067	0,034	0,428	0,828	0,093	0,225
		N	32	32	32	32	32	32	32	32	27	27	27	27
	Day.1_1 Vascular doppler Blood Pressure	Correlation Coefficient	.689**	.596**	1,000	.829**	.393*	.490**	.382*	.504**	.462*	0,338	.683**	.614**
		Sig. (2-tailed)	0,000	0,000		0,000	0,026	0,004	0,031	0,003	0,015	0,085	0,000	0,001
		N	32	32	32	32	32	32	32	32	27	27	27	27
	Day.1_2 Vascular doppler Blood Pressure	Correlation Coefficient	.756**	.801**	.829**	1,000	.448*	.521**	.486**	.575**	.434*	0,367	.667**	.573**
		Sig. (2-tailed)	0,000	0,000	0,000		0,010	0,002	0,005	0,001	0,024	0,060	0,000	0,002
		N	32	32	32	32	32	32	32	32	27	27	27	27
	Day.2_1 Invasive systolic Arteriyal Pressure	Correlation Coefficient	.368*	.389*	.393*	.448*	1,000	.831**	.800**	.754**	.578**	.601**	.525**	.491**
		Sig. (2-tailed)	0,038	0,028	0,026	0,010		0,000	0,000	0,000	0,002	0,001	0,005	0,009
		N	32	32	32	32	32	32	32	32	27	27	27	27
	Day.2_2 Invasive systolic Arteriyal Pressure	Correlation Coefficient	.418*	.462**	.490**	.521**	.831**	1,000	.716**	.894**	.584**	.527**	.546**	.591**
		Sig. (2-tailed)	0,017	0,008	0,004	0,002	0,000		0,000	0,000	0,001	0,005	0,003	0,001
		N	32	32	32	32	32	32	32	32	27	27	27	27
	Day.2_1 Vascular doppler Blood Pressure	Correlation Coefficient	0,253	0,328	.382*	.486**	.800**	.716**	1,000	.800**	.680**	.726**	.563**	.602**
		Sig. (2-tailed)	0,163	0,067	0,031	0,005	0,000	0,000		0,000	0,000	0,000	0,002	0,001
		N	32	32	32	32	32	32	32	32	27	27	27	27
	Day.2_2 Vascular doppler Blood Pressure	Correlation Coefficient	0,309	.377*	.504**	.575**	.754**	.894**	.800**	1,000	.758**	.758**	.667**	.700**
		Sig. (2-tailed)	0,086	0,034	0,003	0,001	0,000	0,000	0,000		0,000	0,000	0,000	0,000
		N	32	32	32	32	32	32	32	32	27	27	27	27
	Day.3_1 Invasive systolic Arteriyal Pressure	Correlation Coefficient	0,149	0,159	.462*	.434*	.578**	.584**	.680**	.758**	1,000	.894**	.721**	.768**
		Sig. (2-tailed)	0,457	0,428	0,015	0,024	0,002	0,001	0,000	0,000		0,000	0,000	0,000
		N	27	27	27	27	27	27	27	27	27	27	27	27
	Day.3_2 Invasive systolic Arteriyal Pressure	Correlation Coefficient	0,097	0,044	0,338	0,367	.601**	.527**	.726**	.758**	.894**	1,000	.616**	.664**
		Sig. (2-tailed)	0,631	0,828	0,085	0,060	0,001	0,005	0,000	0,000	0,000		0,001	0,000
		N	27	27	27	27	27	27	27	27	27	27	27	27
	Day.3_1 Vascular doppler Blood Pressure	Correlation Coefficient	.434*	0,330	.683**	.667**	.525**	.546**	.563**	.667**	.721**	.616**	1,000	.908**
		Sig. (2-tailed)	0,024	0,093	0,000	0,000	0,005	0,003	0,002	0,000	0,000	0,001		0,000
		N	27	27	27	27	27	27	27	27	27	27	27	27
	Day.3_2 Vascular doppler Blood Pressure	Correlation Coefficient	0,350	0,242	.614**	.573**	.491**	.591**	.602**	.700**	.768**	.664**	.908**	1,000
		Sig. (2-tailed)	0,073	0,225	0,001	0,002	0,009	0,001	0,001	0,000	0,000	0,000	0,000	
		N	27	27	27	27	27	27	27	27	27	27	27	27
